# Risk Factors, Prognostic Factors, and Nomogram for Distant Metastasis in Breast Cancer Patients Without Lymph Node Metastasis

**DOI:** 10.3389/fendo.2021.771226

**Published:** 2021-11-24

**Authors:** Yu Min, Xiaoman Liu, Daixing Hu, Hang Chen, Jialin Chen, Ke Xiang, Guobing Yin, Yuling Han, Yang Feng, Haojun Luo

**Affiliations:** Department of Breast and Thyroid Surgery, The Second Affiliated Hospital of Chongqing Medical University, Chongqing, China

**Keywords:** N0 breast cancer, distant metastasis, risk factor, nomogram, cancer-specific survival

## Abstract

**Background:**

Lymph node negative (N0) breast cancer can be found coexisting with distant metastasis (DM), which might consequently make clinicians underestimate the risk of relapse and insufficient treatment for this subpopulation.

**Methods:**

The clinicopathological characteristics of N0 breast cancer patients from the Surveillance, Epidemiology, and End Results (SEER) database between January 2010 and December 2015 were retrospectively reviewed. Multivariate logistic and Cox analyses were used to identify independent risk factors in promoting DM and the 1-, 3-, and 5- year cancer-specific survival (CSS) in this subpopulation.

**Result:**

Seven factors including age (<40 years), tumor size (>10 mm), race (Black), location (central), grade (poor differentiation), histology (invasive lobular carcinoma), and subtype (luminal B and Her-2 enriched) were associated with DM, and the area under curve (AUC) was 0.776 (95% CI: 0.763–0.790). Moreover, T1-3N0M1 patients with age >60 years at diagnosis, Black race, triple-negative breast cancer subtype, no surgery performed, and multiple DMs presented a worse 1-, 3-, and 5-year CSS. The areas under the ROC for 1-, 3-, and 5- year CSS in the training cohort were 0.772, 0.741, and 0.762, respectively, and 0.725, 0.695, and 0.699 in the validation cohort.

**Conclusion:**

The clinicopathological characteristics associated with the risk of DM and the prognosis of female breast cancer patients without lymph node metastasis but with DM are determined. A novel nomogram for predicting 1-, 3-, 5- year CSS in T1-3N0M1 patients is also well established and validated, which could help clinicians better stratify patients who are at a high-risk level for receiving relatively aggressive management.

## Introduction

Breast cancer is currently the most frequent malignancy and one of the leading causes of cancer death in the United States (estimated 279,100 new cases and 42,690 death) (1) and China mainland (estimated 304,000 new cases and 70,000 deaths) (2). Although the long-term survival of patients with breast cancer has been significantly increased in the past years with the application of targeted therapy (3), endocrine therapy (4), and even immunotherapy (5, 6), distant metastasis (DM), as the most common form of recurrence and the main cause (approximately 90%) of death, could reverse this favorable outcome (7, 8). Historically, the “Halsted” hypothesis indicated that the processing steps of breast cancer metastasis were mechanized and orderly, including primary focus enlargement, invasion to the regional lymph nodes, and further metastasis to distant organs *via* the bloodstream. However, subsequent studies on the biological characteristics of breast cancer metastasis have shown that the DM in breast cancer was a non-random process as it allowed circulating tumor cells (CTC) to seed at specific distant tissues, which suggested the metastasis did not require circulation through the lymph system but directly invade the distant organs *via* the bloodstream. Consequently, the CTC analysis technique has become a novel utility tool for predicting the prognosis of breast cancer patients, which could provide better treatment guidance for clinicians (9, 10).

Indeed, as a key component of tumor stage classification, the status of the regional lymph nodes plays an important role in predicting the biological aggressiveness and propensity to spread in patients with breast cancer (11, 12). Some scholars believe that regional nodal disease may precede metastatic dissemination (11). Therefore, after surgery, patients with negative lymph node status could remain a favorable outcome, and only a small fraction of them need adjuvant therapy during the postoperative follow-up (11). Additionally, reviewing the recent literature, negative lymph node status was frequently referred to as the “control group” in the study when scholars aimed to explore the risk factors of DM (13–16). Patients with negative lymph node status were more likely to be assigned to the low-risk group. However, one thing that cannot be ignored was that there were still a considerable proportion of patients screened out having DM but negative lymph node status (17). The insufficient adjuvant therapy and management for this population might increase the risk of relapse in those lymph-node-negative (N0) patients with multiple risk factors. And clinicians may underestimate the risk of relapse and make insufficient treatment for N0 patients with breast cancer.

Therefore, it is equally important to identify the independent risk factors of DM in this particular subpopulation, which would not only help oncologists to begin tailoring treatment strategies to patients but also encourage researchers to investigate the underlying molecular mechanisms in breast cancer metastasis. Although some scholars have made efforts on evaluating the DM in lymph node negative primary breast cancer *via* evaluating the gene expression profiles and the integration of proliferation and immunity (17, 18), whether there was a different clinical pattern between DM and non-DM patients without lymph node involvement was still unclear.

In the present study, we aimed to extract the potential risk clinicopathological factors in promoting DM of N0 primary breast cancer, which would fill the gap in identifying high-risk subgroups. Besides, we also evaluated the cancer-specific survival (CSS) in this subpopulation and further developed a novel predictive model to provide quantitative predictions on the outcome for N0 patients with DM. More aggressive treatment modalities and active surveillance may be justified in high-risk subgroups of patients.

## Materials and Methods

### Data Source

This is an observational retrospective cohort study. As a result, the data we analyzed were extracted from a large population-based (Surveillance, Epidemiology, and End Results, SEER, derived from the 18 cancer registries) research program, which included approximately 28% of the U.S. population and various ethnic groups. The medical records collection and analysis were performed by two study researchers, working independently to decrease the selection bias. The reporting of this study followed the guidelines of the Strengthening the Reporting of Observational Studies in Epidemiology (STROBE) statement (19).

**Figure 1:**
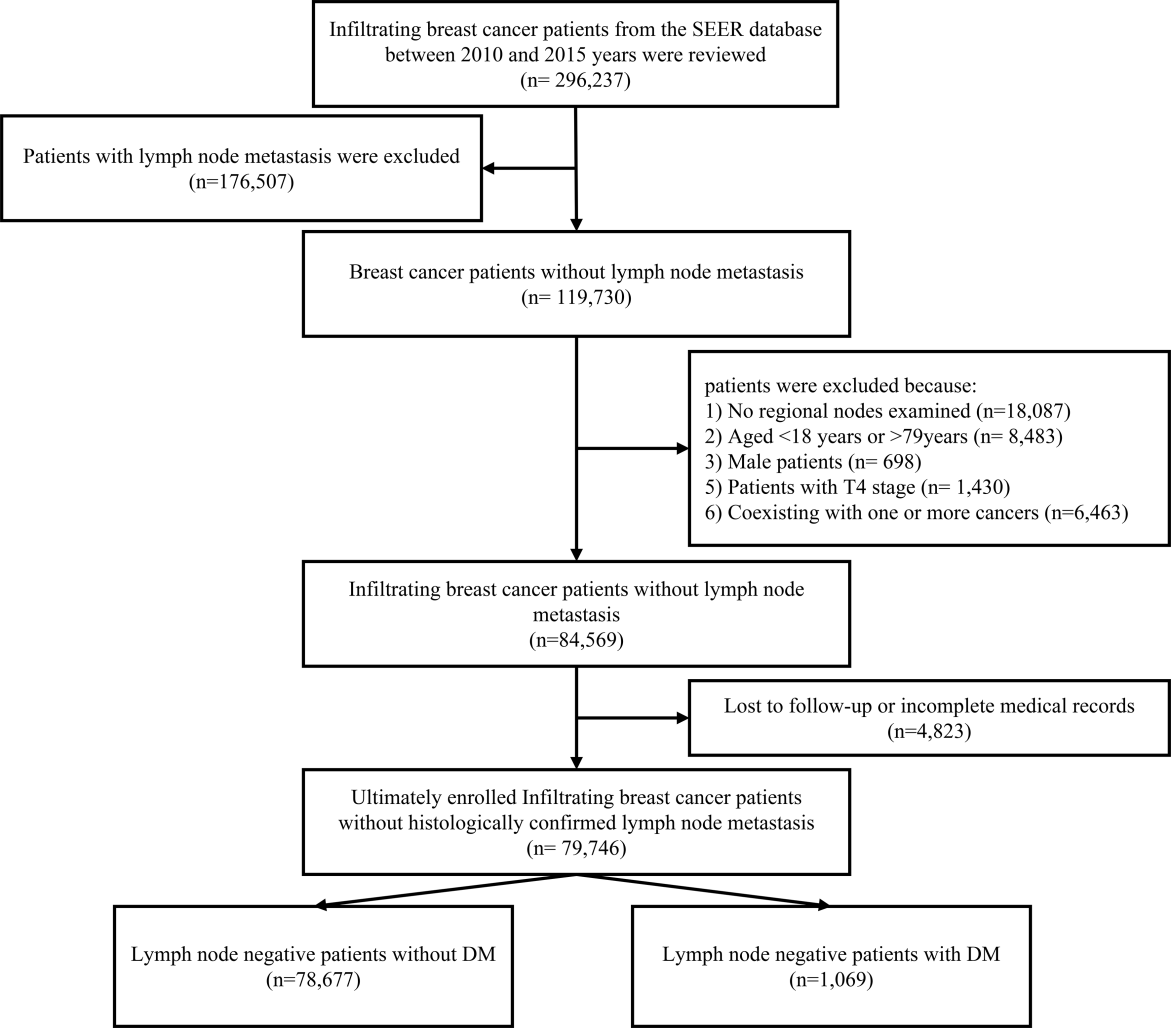
The patients’ selection processing. T4, invasion to the chest wall/skin and inflammatory carcinoma; DM, distant metastasis.

Patients who met the following criteria were included: (1) female patients with histological confirmed invasive breast cancer; (2) aged at diagnosis between 18 and 79 years; (3) pathological confirmed negative lymph node status; (4) diagnosed between 2010 and 2015 years; (5) the histology types of breast cancer were infiltrating ductal carcinoma (IDL), infiltrating lobular carcinoma (ILC), and infiltrating ductal mixed lobular carcinoma (IDLC). Patients with T4 (invasion to the chest wall/skin and inflammatory carcinoma) primary site, no regional nodes examined, coexisting with one or more cancers, lost to follow-up, or incomplete medical records were excluded during the patients’ selection process (**Figure 1**).

### Variable Evaluation and Definition

According to the requirement of establishing sample size of multivariate linear regression equation, the sample size in the present study should be at least 10 times of the number of independent variables in the equation. Thus, after excluding the unqualified cases, there were 79,746 female patients with invasive breast cancer enrolled in this study. They were assigned to explore the risk factors in promoting the DM in N0 breast cancer. Besides, for predicting 1-, 3-, and 5-year CSS, the N0 patients with DM between 2010 and 2015 years were randomly divided into a training group and validation group at a ratio of 7:3 *via* the “R” program.

We selected the variables on the basis of their associations with the outcomes of interest. Specifically, the following clinicopathological characteristics were collected and transformed into categorical variables: age (≥20 and <40 years; ≥40 and <60 years; ≥60 and <80 years), race (White, Black, Asian or Pacific Islander, and American Indian/Alaska Native), laterality (right and left origin of primary), stage (I, II, IV derived from AJCC staging system 7th edition), grade (well differentiated, moderately differentiated, poorly differentiated, and undifferentiated), location (central, outer, inner, overlapping, and axillary of breast), histological type (IDC, ILC, and IDLC), ICD-O-3 codes (8500/3, 8520/3, 8521/3, and 8522/3), breast cancer subtype [Luminal A: hormonal receptor (HR)+/HER2−, Luminal B: HR+/HER2+, Triple-negative: HR−/HER2−, Her-2 enriched: HR−/HER2+], primary tumor size (T_1mic_: >0 and ≤1 mm; T_1a_: >1 and ≤5 mm; T_1b_: >5 and ≤10 mm; T_1c_: >10 and ≤20 mm; T_2_: >20 and ≤50 mm; T_3_: >50 mm), DM at meet (bone, liver, lung, brain, and multiple DM); surgery; cause-specific death, and 60 survival months (more than 0 days of survival).

### Statistical Analysis

The primary endpoint of this study was DM and 1-, 3-, and 5-year CSS probability. The univariate and multivariate logistic analyses were used for identifying the potential independent clinical risk factors in promoting DM of lymph node negative patients. And the univariate and multivariate Cox regression analyses were performed to find out the prognostic factors of CSS in patients with DM. The analyses were conducted *via* IBM SPSS (version 25.0). A two-tailed P-value of <0.05 was defined as the criterion for variable deletion when performing backward stepwise selection. The nomogram, calibration curve, and Kaplan-Meier analysis were constructed and plotted based on the results of the multivariate Cox regression analysis *via* using the “survival,” “rms,” “survminer,” and “foreign” packages of the R software (R Foundation, Vienna, Austria, version 3.5.2, http://www.r-project.org). Harrell’s C-index is calculated to assess the discrimination performance of the present nomogram.

## Result

### Clinicopathological Characteristics of Patients With Negative Lymph Node Status

Generally, between the years 2010 and 2015, a total of 79,746 female patients with invasive breast cancer were enrolled in this study with a median age of 61 years (range: 20–79 years) at diagnosis and a median follow-up time of 51 months (range: 0–95 months). There were 1,069 cases (1.34%) identified coexisting with DM in the N0 patients, in which 748 cases were observed in the training cohort and 321 cases were in the validation cohort (**Table 1**). Specifically, the most frequent metastasis site was bone, which made up 327 cases (43.72%) and 150 cases (46.73%) of the DM patients in the training and validation cohorts. Notably, 385 (36.01%) patients suffered from multiple DMs. And almost 70.63% (755/1,069 cases) of patients with DM did not receive surgery for the primary tumor.

**Table 1:** Clinicopathological characteristics of female patients with negative lymph node status but distant metastasis in training and validation cohorts.

Characteristics	No. (%) of patients		
	Initial cohort (n = 1,069)	Training cohort (n = 748)	Validation cohort (n = 321)
**Age**			
≥20 and <40	78 (7.3)	53 (7.09)	25 (7.79)
≥40 and <60	416 (38.91)	289 (38.64)	127 (39.56)
≥60 and <80	575 (53.79)	406 (54.28)	169 (52.65)
**Race**			
White	866 (81.01)	605 (80.88)	261 (81.31)
Black	140 (13.10)	99 (13.24)	41 (12.77)
**^※^** Other	63 (5.89)	44 (5.88)	19 (5.92)
**Location**			
Nipple	4 (0.37)	3 (0.40)	1 (0.31)
Central	75 (7.01)	50 (6.68)	25 (7.79)
Upper-inner	144 (13.47)	93 (12.43)	51 (15.89)
Lower-inner	61 (5.71)	42 (5.61)	19 (5.92)
Upper-outer	385 (36.01)	268 (35.83)	117 (36.45)
Lower-outer	97 (9.07)	71 (9.49)	26 (8.10)
Axillary	11 (3.43)	8 (1.07)	3 (0.93)
Overlapping	292 (28.34)	213 (28.48)	79 (24.61)
**^&^Grade**			
I	138 (12.91)	96 (12.83)	42 (13.08)
II	535 (50.04)	386 (51.60)	149 (46.42)
III/IV	396 (37.04)	266 (35.56)	130 (40.50)
**Laterality**			
Right	496 (46.40)	356 (47.59)	140 (43.61)
Left	573 (53.60)	392 (52.41)	181 (56.39)
**Histology**			
IDC	870 (81.39)	610 (81.55)	260 (81.00)
ILC	142 (13.28)	98 (13.10)	44 (13.71)
IDLC	57 (5.33)	40 (5.35)	17 (5.29)
**Tumor size**			
T_1mic_	1 (0.01)	0 (0.00)	1 (0.31)
T_1a_	20 (1.89)	15 (2.00)	5 (1.56)
T_1b_	67 (6.27)	47 (6.28)	20 (6.23)
T_1c_	240 (22.45)	164 (21.92)	76 (23.68)
T_2_	605 (56.59)	436 (58.30)	169 (52.65)
T_3_	136 (12.72)	86 (11.50)	50 (15.58)
**M status**			
M_1_-bone	477 (44.62)	327 (43.72)	150 (46.73)
M_1_-liver	95 (8.89)	67 (8.96)	28 (8.72)
M_1_-lung	102 (9.54)	73 (9.76)	29 (9.03)
M_1_-brain	10 (0.94)	7 (0.94)	3 (0.93)
M_1_-multiple	385 (36.01)	247 (33.02)	111 (34.58)
**Subtype**			
Luminal A	693 (64.83)	493 (65.91)	200 (62.30)
Luminal B	175 (16.37)	118 (15.78)	57 (17.76)
TNBC	134 (12.54)	92 (12.30)	42 (13.08)
Her-2 enriched	67 (6.27)	45 (6.02)	22 (6.85)
**Surgery**			
Not performed	755 (70.63)	534 (71.39)	221 (68.85)
Performed	314 (29.37)	214 (28.61)	100 (31.15)

^※^Other: defined as the Asian/Pacific Islander and American Indian/Alaska Native; ^＆^Grade: I, well differentiated; II, moderately differentiated; III/IV, poorly differentiated and undifferentiated.

IDC, invasive ductal carcinoma; ILC, invasive lobular carcinoma; IDLC, invasive ductal mixed with lobular carcinoma; TNBC, triple-negative breast cancer; Her-2, human epidermal growth factor receptor-2.

### Univariate and Multivariate Logistic Analyses of the Risk Factors of DM

To investigate the potential clinical factors associated with the risk of DM in female breast cancer with negative lymph node status, the logistics analysis was performed. During the univariate logistic analysis, age at diagnosis (p<0.0001), tumor size (p<0.0001), race (p<0.0001), tumor location (p<0.0001), grade (p<0.0001), histology (p<0.0001), and subtype (p<0.0001) were identified to be significantly associated with DM. Thus, we incorporated seven clinicopathological factors into the multivariate logistic analysis and further obtained a good AUC of 0.776 (95% CI: 0.763–0.790) (**Supplementary Figure S1**) in predicting the risk of DM in female patients with negative lymph node status. Specifically, the results presented that tumor size >10 mm [>10 maximum diameter ≤20 mm: hazard ratio (HR)= 2.28, 95% confidence interval (CI): 1.78–2.92; >20 maximum diameter ≤50mm: HR=9.46, 95% CI: 7.51–11.92; maximum diameter >50 mm: HR= 19.12, 95% CI: 14.44–25.33; <0.0001], Black race (HR=1.12, 95% CI: 0.93–1.35, p<0.0001), moderate grade (HR=1.57, 95% CI: 1.29–1.90, p<0.0001), ILC (HR=1.31, 95% CI: 1.08–1.58, p<0.0001), and subtype (luminal B: HR=1.54, 95% CI: 1.29–1.84; Her-2 enriched: HR=1.46, 95% CI: 1.12–1.92; p<0.0001). On the contrary, elderly age (≥40 age <60 years: HR=0.70; 95% CI: 0.54–0.89; ≥60 age <80 years: HR= 0.84; 95% CI: 0.65–1.08; p=0.002), Asian/Pacific Islander and American Indian/Alaska Native race (HR=0.52, 95% CI: 0.40–0.67, p<0.0001), and tumor location (inner location: HR=0.57, 95%CI: 0.44–0.75; outer location: HR= 0.64, 95% CI: 0.50–0.82; axillary and overlapping location: HR= 0.71, 95% CI: 0.55–0.92; p<0.0001, respectively) were determined to be the protective factors in DM (**Table 2**).

**Table 2:** Univariate and multivariate logistic regression analyses of clinical variables correlated with distant metastasis in female breast cancer with negative lymph node status.

Variables	Subgroup	Univariable		Multivariable	
		Hazard ratio	*P*	Hazard ratio	*P*
**Age (year)**	≥20 and <40	Reference	**<0.0001**	Reference	**0.002**
	≥40 and <60	0.49 (0.38–0.63)		0.70 (0.54–0.89)	
	≥60 and <80	0.52 (0.41–0.67)		0.84 (0.65–1.08)	
**Tumor size (mm)**	>0 and ≤10	Reference	**<0.0001**	Reference	**<0.0001**
	>10 and ≤20	2.37 (1.86–3.03)		2.28 (1.78–2.92)	
	>20 and ≤50	10.09 (8.06–12.63)		9.46 (7.51–11.92)	
	>50	21.79 (16.60–28.61)		19.12 (14.44–25.33)	
**Race**	White	Reference	**<0.0001**	Reference	**<0.0001**
	Black	1.31 (1.09–1.57)		1.12 (0.93–1.35)	
	**^※^** Other	0.57 (0.44–0.74)		0.52 (0.40–0.67)	
**Location**	**^＆^** Central	Reference	**<0.0001**	Reference	**<0.0001**
	Inner	0.47 (0.36–0.61)		0.57 (0.44–0.75)	
	Outer	0.55 (0.43–0.70)		0.64 (0.50–0.82)	
	**^¶^** Other	0.62 (0.48–0.80)		0.71 (0.55–0.92)	
**Grade**	Well	Reference	**<0.0001**	Reference	
	Moderate	2.34 (1.94–2.83)		1.57 (1.29–1.90)	**<0.0001**
	Poor	2.68 (2.21–3.26)		1.22 (0.98–1.52)	
**Laterality**	Right	Reference	0.068	**/**
	Left	1.11 (0.99–1.26)		
**Histology**	IDC	Reference	**<0.0001**	Reference	**<0.0001**
	ILC	1.71 (1.43–2.05)		1.31 (1.08–1.58)	
	IDLC	1.07 (0.82–1.41)		0.99 (0.75–1.31)	
**Subtype**	Luminal A	Reference		Reference	
	Luminal B	1.96 (1.66–2.32)	**<0.0001**	1.54 (1.29–1.84)	**<0.0001**
	TNBC	1.30 (1.08–1.56)		0.89 (0.72–1.10)	
	Her-2	1.99 (1.54–2.56)		1.46 (1.12–1.92)	

**^※^** Other: defined as the Asian/Pacific Islander and American Indian/Alaska Native; **^＆^** Central: central portion of breast combined with nipple; **^¶^** Other: axillary and overlapping of the breast.

IDC, invasive ductal carcinoma; ILC, invasive lobular carcinoma; IDLC, invasive ductal mixed with lobular carcinoma; TNBC, triple-negative breast cancer; Her-2, human epidermal growth factor receptor-2.

Bold values indicate statistical significance (p < 0.05).

### Univariate and Multivariate Cox Analyses of the Risk Factors of CSS

To identify the independent risk factors of 1-, 3-, and 5-year CSS in women with negative lymph node status but DM during the follow-up, only significant factors from univariate Cox regression analysis were further applied into multivariate Cox regression analysis. During the univariate Cox regression analysis, age (*p*=0.017), race (*p*<0.0001), grade (*p*=0.004), subtype (*p*<0.0001), tumor size (p=0.027), surgery (*p*<0.0001), and metastasis site (p<0.0001) were identified to be the predictive factors. Additionally, elderly age (≥60 age <80 years: HR= 1.58; 95% CI: 1.03–2.44; p=0.015), black race (HR=1.64, 95% CI: 1.24–2.16, p=0.002), TNBC (HR=2.77, 95% CI: 2.02–3.80, p<0.0001), and metastasis site (liver: HR=2.01, 95% CI: 1.36–2.90; multiple sites: HR=2.13, 95% CI: 1.69–2.68, p<0.0001) were regarded as the independent risk factors of CSS in this subpopulation (**Table 3**). However, the tumor size (p=0.123) and differentiation grade (p=0.101) were not determined to be statistically significant.

**Table 3:** Univariate and multivariate Cox regression analyses of predictive variables correlated with CSS in IV stage female breast cancer with negative-lymph node status.

Variables	Subgroup	Univariable		Multivariable	
		Hazard ratio	*P*	Hazard ratio	*P*
**Age (year)**	≥20 and <40	Reference	**0.017**	Reference	**0.015**
	≥40 and <60	1.29 (0.84–2.00)		1.21 (0.78–1.88)	
	≥60 and <80	1.63 (1.07–2.48)		1.58 (1.03–2.440	
**Race**	White	Reference	**<0.0001**	Reference	**0.002**
	Black	1.72 (1.31–2.24)		1.64 (1.24–2.16)	
	**^※^** Other	0.86 (0.54–1.36)		0.89 (0.56–1.44)	
**Location**	**^＆^** Central	Reference	0.991	**/**
	Inner	1.06 (0.67–1.65)		
	Outer	1.02 (0.68–1.54)		
	**^¶^** Other	1.01 (0.66–1.56)		
**Grade**	I	Reference	**0.004**	Reference	0.101
	II	1.30 (0.92–1.82)		1.35 (0.95–1.91)	
	III/IV	1.68 (1.19–2.38)		1.52 (1.03–2.23)	
**Histology**	IDC	Reference	0.821	**/**
	ILC	1.07 (0.80–1.43)		
	IDLC	0.92 (0.58–1.44)		
**Laterality**	Right	Reference	0.816	**/**
	Left	1.00 (0.82–1.21)		
**Subtype**	Luminal A	Reference	**<0.0001**	Reference	**<0.0001**
	Luminal B	0.92 (0.69–1.23)		0.79 (0.58–1.07)	
	TNBC	2.52 (1.92–3.30)		2.77 (2.02–3.80)	
	HER2	1.37 (0.90–2.06)		1.29 (0.82–2.01)	
**Tumor size (mm)**	>0 and ≤10	Reference	**0.027**	Reference	0.123
	>10 and ≤20	0.66 (0.46–0.96)		0.78 (0.54–1.14)	
	>20 and ≤50	0.63 (0.45–0.88)		0.72 (0.51–1.00)	
	>50	0.54 (0.34–0.83)		0.602 (0.38–0.93)	
**Surgery**	No	Reference	**<0.0001**	Reference	**<0.0001**
	Yes	0.51 (0.40–0.64)		0.39 (0.30–0.51)	
**M status**	Bone	Reference	**<0.0001**	Reference	**<0.0001**
	Liver	2.20 (1.55–3.12)		2.01 (1.396–2.90)	
	Lung	1.34 (0.92–1.95)		1.06 (0.72–1.57)	
	Brain	1.84 (0.68–4.97)		1.44 (0.528–3.93)	
	^*^ Multiple	2.31 (1.85–2.89)		2.13 (1.69–2.68)	

**^※^** Other: defined as the Asian/Pacific Islander and American Indian/Alaska Native; **^＆^** Central: central portion of breast combined with nipple; **^¶^** Other: axillary and overlapping of the breast; ^*^ Multiple: two or more distant metastasis sites.

IDC, invasive ductal carcinoma; ILC, invasive lobular carcinoma; IDLC, invasive ductal mixed with lobular carcinoma; TNBC, triple-negative breast cancer; HER2, human epidermal growth factor receptor-2.

Bold values indicate statistical significance (p < 0.05).

**Figure 2:**
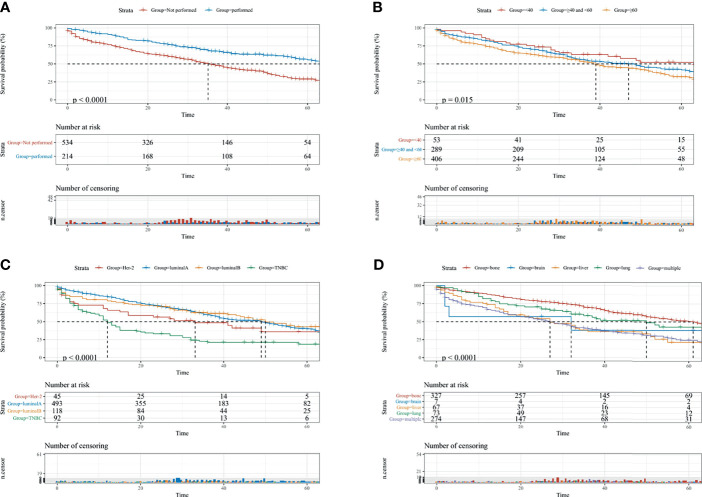
The KM survival curves for predicting the CSS of lymph-node-negative women with DM. **(A)** Surgical intervention; **(B)** age at diagnosis; **(C)** breast subtype; **(D)** metastasis sites. CSS, cancer-specific survival; Multiple, two or more distant metastasis sites.

Furthermore, to actuarially estimate the survival probability and cumulative hazard in patients with different variables, five factors (p ≤ 0.05) from multivariate analysis in Cox proportional hazard model were used to plot the Kaplan-Meier survival curves; namely, a significant decrease in survival probability was observed in patients without surgery performed (1-, 3-, and 5-year CSS rate: 73.8, 48.9, and 28.8%, respectively, **Figure 2A**), age ≥60 (1-, 3-, and 5-year CSS rate: 74.2, 52.5, and 32.1%, respectively, **Figure 2B**), TNBC subtype (1-, 3-, and 5-year CSS: 48.7, 21.4, and 18.7%, respectively, **Figure 2C**), and DM to multiple sites (1-, 3-, and 5-year CSS: 66.6, 40.1, and 24.4%, respectively, **Figure 2D**), as well as Black race (1-, 3-, and 5-year CSS: 61.5, 35.8, and 24.6%, respectively, **Supplementary Figure S2**) were all associated with the survival probability.

### Predictive Nomogram Construction and Validation

**Figure 3:**
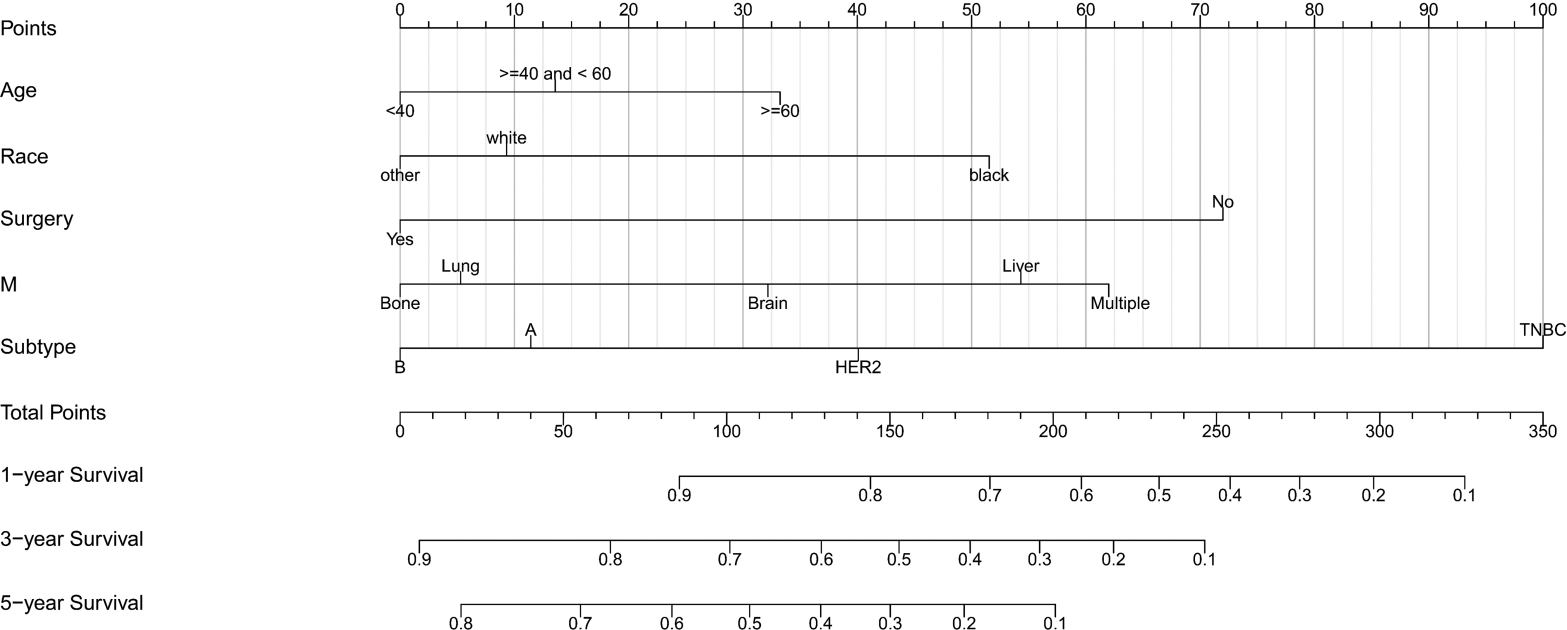
Nomogram for predicting the 1-, 3-, and 5-year CSS in lymph-node-negative women with DM. Other: defined as the Asian/Pacific Islander and American Indian/Alaska Native. CSS, cancer-specific survival; A, Luminal A; B, Luminal B; TNBC, triple-negative breast cancer; HER2, Her-2 enriched.

**Figure 4:**
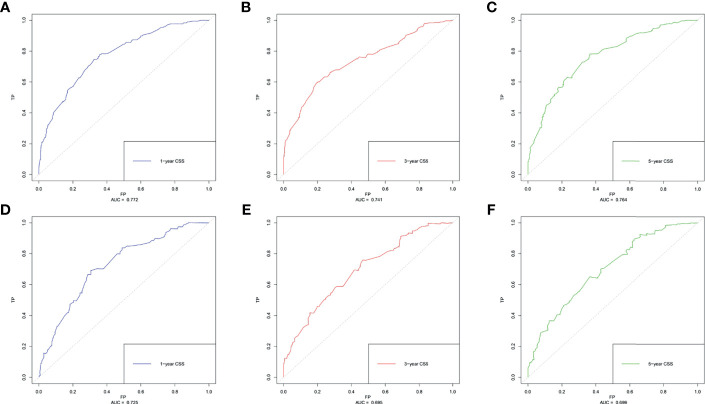
The receiver operating characteristics (ROC) curve and area under the ROC curve (AUC). **(A)** Predicting 1-year CSS in the training cohort; **(B)** predicting 3-year CSS in the training cohort; **(C)** predicting 5-year CSS in the training cohort; **(D)** predicting 1-year CSS in the validation cohort; **(E)** predicting 3-year CSS in the validation cohort; **(F)** predicting 5-year CSS in the validation cohort.

**Figure 5:**
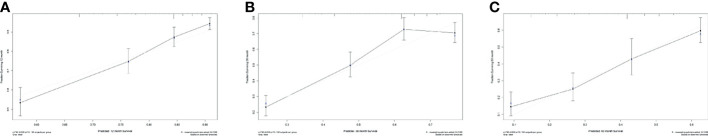
Calibration curves for evaluating the accuracy of the nomogram. **(A)** 1-year CSS in lymph-node-negative women with DM, **(B)** 3-year CSS in lymph-node-negative women with DM, and **(C)** 5-year CSS in lymph-node-negative women with DM. The solid black line represents the performance of the nomogram, of which the closer fit to the gray line represents the better prediction of the nomogram we constructed. CSS, cancer-specific survival.

Based on the multivariate Cox regression analysis, five variables including age at diagnosis, race, surgery performed, distant metastasis site, and tumor subtype were extracted for constructing the nomogram for predicting the 1-, 3-, and 5- ear CSS in patients with negative lymph node status but DM at meet (**Figure 3**). Each factor represented a score on the points scale, and the total point could be calculated by adding up all the specific values from an individualized patient. The C-index of the nomogram reached 0.694, which represented relatively favorable discrimination (the specific value of each variable was calculated in **Table 4**). In the training cohort, the AUC of each 1-, 3-, and 5-year CSS ROC was 0.772, 0.741, and 0.762 with a cutoff value of 185, 191, and 151, respectively, which indicated a satisfying prediction ability (**Figures 4A–C**). Moreover, the established nomogram was validated by an internal validation cohort with 321 cases. The results in the validation cohort also presented good discrimination with an AUC of 0.725 in predicting the 1-year CSS (**Figure 4D**), an AUC of 0.695 in predicting the 3-year CSS (**Figure 4E**), and an AUC of 0.699 in predicting the 5-year CSS (**Figure 4F**), respectively. Besides, to examine the discrimination of the proposed nomogram, the patients in the training set were categorized into four groups based on the total points obtained from the nomogram. The KM curve presented good discrimination in identifying the high-risk population (**Supplementary Figure S3**). To further evaluate the accuracy of the nomogram, the calibration curves for the probability of CSS presented a high agreement between 1-, 3-, and 5-year predictions of the nomogram (**Figure 5**).

**Table 4:** The specific value of clinicopathological factors in the nomogram in the training cohort.

Characteristics	Score
**Age**	
≥20 and <40	0
≥40 and <60	14
≥60 and <80	33
**Race**	
White	9
Black	52
**^※^** Other	0
**M status**	
M_1_-bone	0
M_1_-liver	54
M_1_-lung	5
M_1_-brain	32
M_1_-**^＆^** multiple	62
**Subtype**	
Luminal A	11
Luminal B	0
TNBC	100
Her-2 enriched	40
**Surgery**	
No	72
Yes	0
**Total point for 1-year CSS**	
0.1	326
0.2	298
0.3	275
0.4	254
0.5	232
0.6	209
0.7	181
0.8	144
0.9	86
**Total point for 3-year CSS**	
0.1	246
0.2	218
0.3	196
0.4	175
0.5	153
0.6	129
0.7	101
0.8	64
0.9	6
**Total point for 5-year CSS**	
0.1	201
0.2	173
0.3	150
0.4	129
0.5	107
0.6	83
0.7	55
0.8	19

**^※^** Other: defined as the non-Hispanic Asian/Pacific Islander and American Indian/Alaska Native; **^＆^** Multiple: two or more distant metastasis sites.

TNBC, triple-negative breast cancer; HER2, human epidermal growth factor receptor-2; CSS, cancer-specific survival.

## Discussion

Nowadays, breast cancer has become the most frequent malignancy among women worldwide (1, 2, 20). While the overall survival (OS) rate in breast cancer patients has improved with the help of early-detection and multiple treatment modalities, patients who were diagnosed with DM at presentation still underwent a worse prognosis. In the last decades, great advances have been achieved in understanding and detecting breast cancer metastasis. Breast cancer was no longer regarded as a locoregional but systemic disease with an inherent feature of metastasis (8). There is no doubt that regional lymph node involvement is one of the important predictive factors in breast cancer DM. Even some scholars suggested and validated that regional node metastasis could precede metastatic dissemination (11). Notably, a considerable number of N0 patients were observed occurring *de novo* DM. With the wide application of circulating tumor cell (CTC) analysis, many scholars recognized that the DM was considered triggered by hematogenous spread of CTCs, rather than by lymphatic or direct intracavitary spread, which possibly occurred by a different mechanism. For this reason, breast cancer patients without regional lymph node metastasis but distant organ invasion would be the objects for exploring the underlying mechanisms.

However, only a few previous studies could be reviewed in predicting the risk factors and the prognosis of N0 patients (17, 18, 21). Herein, we provided a new insight in exploring whether there was a significant difference between N0 patients with DM or not, and the prognosis of those patients with DM was also evaluated. In this study, the incidence rate of DM in N0 patients was about 1.34% (1,069/79,746). Several clinicopathological factors including age at diagnosis, tumor size, race, tumor location, differentiation grade, histology, and subtype were significantly associated with DM. Younger patients (especially <40 years) have nearly twice the risk of DM than elderly patients, which was in accordance with Sabiani’s report (22). Consistent with previous studies on evaluating the risk factors of DM in patients with invasive breast cancer, patients with tumor size (>10 mm), ILC, estrogen receptor (ER), and progesterone receptor (PR) as well as Her-2-positive subtype, and Black race (**Supplementary Figure S2**) presented a higher risk of DM (12, 14, 21). In terms of the tumor location, it has been determined that tumor location was significantly associated with the regional lymph node metastasis, especially when the tumor originated from the nipple and central location as well as overlapping of the breast (23–26). We took it a step further that the nipple and central tumor locations were identified had a higher risk of DM (p<0.0001) in N0 women. Despite that we have discovered seven independent risk factors associated with DM in N0 patients, further studies are needed to verify the underlying molecular mechanisms in promoting this complex process.

Notably, some researchers have conducted to explore the risk factors of DM and the prognosis of patients with DM at presentation (7, 13, 15, 16, 27). For instance, Rosa Mendoza determined that tumor stage, primary tumor size, and lymph node involvement were the major predictors of DM in adult breast cancer (14). Besides, the Black race and Her-2-enriched subtype were also identified as the risk factors of DM in a recent study (28–30). In the present study, we explored the prognostic factors of 1-, 3-, and 5-year CSS among 748 N0 patients with DM. Although the N0 women at a young age were more likely to have DM, compared with elderly women, the young population, however, had better long-term outcomes than the elderly population (HR= 1:1.58; 95% CI: 1.03–2.44). This result was consistent with one recent large population-based epidemiological study in Brazil that young women had a lower rate of modality (31). On the contrary, in another study by Sabiani and colleagues, they concluded that patients at a young age (<35 years) had the lower estimated disease-free survival (DFS) and OS rate (22). These discrepancies might be due to the differences in sample size and patient inclusion criteria. For example, all included patients in their study were under 50 years old, while the patients in ours were at the age between 20 and 79 years old combined with negative lymph node status. Additionally, we determined five independent risk factors in the poor CSS probability of N0 patients with DM.

Moreover, the role of surgical treatment for the primary focus is regarded as a palliative surgery for patients with DM, and whether patients with DM can benefit from it remains controversial (32–35). One meta-analysis derived from two randomized controlled trials presented that there was no final conclusion about the role of surgery performed in breast cancer patients with DM at presentation (35). With further exploration, some studies, including the present study, found that locoregional surgery would improve the CSS and OS outcomes of metastatic breast cancer (15, 32, 34, 36). Indeed, there were still many questions on the discussion of the timing, type, and extension of the surgical procedures, which needed to be addressed in future works (33). Noticeably, compared with the previous study on evaluating the prognostic factors for patients with DM, primary tumor size (p=0.123) and grade differentiation (p=0.101) were not significantly associated with the CSS in the N0 population. In a similar studied population, Yu and his colleagues (29) determined that the larger tumor size was non-linear with the DM in N0 patients. They consequently believed the primary tumor biological features rather than the accumulated metastatic ability during tumor evolution likely determined the potential of distant dissemination, which indicated the indolent biological characteristics of the tumor. Accordingly, our results support this hypothesis but need further evaluation.

To visualize and more intuitively present the prognostic factors we determined for clinical use, the nomogram model was subsequently plotted. Markedly, in the nomogram, the breast cancer subtype accounted for a major part of the scoring system. Referencing similar nomograms for evaluating the prognostic of breast cancer (37, 38), the TNBC subtype was determined to yield the highest score. Consequently, the clinicians could obtain the risk coefficient in 1-, 3-, and 5-year CSS probability. Compared with other recent works on evaluating the 3- and 5-year CSS in breast cancer women with bone metastasis, the C-index of the present nomogram was 0.694, which was higher than Liu’s (0.660) (16) and very close to the C-index of nomograms developed by Wang (0.705) (15) and Zhao (0.723) (37), confirming the promising discrimination of our model. To evaluate the accuracy of the nomogram, an independent cohort was subsequently used for validation. Expectedly, the AUC of the 1-, 3-, and 5-year CSS predicting ROC in the validation cohort reached 0.725, 0.695, and 0.699, respectively, which further proved the utility of our model to be applied to access the long-term CSS in this subpopulation. Besides, compared with the study of Wang and colleagues (17), the number of N0 patients in the training cohort of the present study was considerably large (748 *vs* 286). Different from previous studies of N0 patients with a focus on gene expression profiles (17, 18), we provide a new insight in accessing the individual risk of DM and long-term CSS probability in N0 patients based on the clinicopathological characteristics. For instance, a 65-year-old black woman was diagnosed with HER2+ tumor, with only bone involvement. This patient would have a total of 125 points and an estimated 1-year, 3-year, and 5-year CSS of 84, 62, and 41% probability after surgery.

Alternatively, this study has some limitations that have to be addressed in the future works. First, this is a retrospective study in which selection bias inevitably exists. Second, while the SEER database contains approximately 28% population-based cancer registration data, some significant confounding prognostic factors including but not limited to Ki-67 index (39), BRCA1- and BRCA2-related mutation (40, 41), as well as high 21-Gene Recurrence Score (21-GRS) (42), which have been proved to be related to worse survival in patients with breast cancer, are unavailable in the SEER database. Third, further information about adjuvant management of these patients was not reported in the present study, as these data were limited in the SEER database. Consequently, future works are supposed to fill this gap to get robust clinical evidence. Besides, with the technical advances in multidisciplinary management, the CSS in patients with breast cancer would increase in the future, which could influence the predictive ability of the model. Lastly, another weakness of this study is the lack of an external validation cohort, which limits further enforcing the reliability and clinical application of the nomogram. Thus, more external validation cohorts from multicenter and countries are urgently demanded to further evaluate the feasibility of our nomogram.

## Conclusion

In summary, this study first identified the potential risk clinicopathological characteristics of DM in N0 patients and the prognostic factors in patients with DM at presentation. N0 patients with younger age at diagnosis, larger tumor size, central tumor location, Black race, poorer differentiated grade, ILC, and luminal B subtype have the highest risk of DM, which could help clinicians to avoid underestimating the risk of DM and subsequent undertreatment in N0 patients. However, DM patients with elderly age at diagnosis, TNBC subtype, and multiple metastasis sites have the worst prognosis. Besides, the novel validated nomogram could help clinicians to better stratify patients who are at high risk of cancer-specific death for receiving relatively aggressive treatment and management. Meanwhile, we propose more external validation to further strengthen our findings.

## Data Availability Statement

The original contributions presented in the study are included in the article/**Supplementary Material**. Further inquiries can be directed to the corresponding authors.

## Ethics Statement

Ethical approval was waived by the local Ethics Committee of the Chongqing Medical University in view of the retrospective nature of the study and all the procedures being performed were part of the routine care.

## Author Contributions

All authors contributed to conception and design of the study. YM, XL, and HC organized the database. YM, YF, JC, KX, GY, and YH performed the statistical analysis. All authors wrote the first draft of the manuscript. All authors wrote sections of the manuscript. All authors contributed to the article and approved the submitted version.

## Funding

This work was supported in part by the National Natural Science Foundation of China (NSFC No. 82072938) for HL.

## Conflict of Interest

The authors declare that the research was conducted in the absence of any commercial or financial relationships that could be construed as a potential conflict of interest.

## Publisher’s Note

All claims expressed in this article are solely those of the authors and do not necessarily represent those of their affiliated organizations, or those of the publisher, the editors and the reviewers. Any product that may be evaluated in this article, or claim that may be made by its manufacturer, is not guaranteed or endorsed by the publisher.

## Supplementary Material

Supplementary Figure 1 The ROC curve and AUC of the risk factors in promoting distant metastasis in lymph-node-negative women.

Supplementary Figure 2 The KM survival curves for predicting the CSS of lymph-node-negative women with DM according to race.

Supplementary Figure 3 The KM survival curves for predicting the CSS in the different risk population, based on the risk stratification of the nomogram.

## Abbreviations

DM, distant metastasis; CSS, cancer-specific survival; N0, lymph node negative; ROC, receiver operating characteristic curves; CTCs, circulating tumor cells; IDL, infiltrating ductal carcinoma; ILC, infiltrating lobular carcinoma; IDLC, infiltrating ductal mixed lobular carcinoma; OS, overall survival; CSS, cancer-specific survival; ER, estrogen receptor; PR, progesterone receptor.
