# Supplementary figures and images for: Risk Factors, Prognostic Factors, and Nomogram for Distant Metastasis in Breast Cancer Patients Without Lymph Node Metastasis
Source: Front Endocrinol (Lausanne). 2021 Nov 24;12:771226. doi: 10.3389/fendo.2021.771226 (PMC8653828; doi:10.3389/fendo.2021.771226)

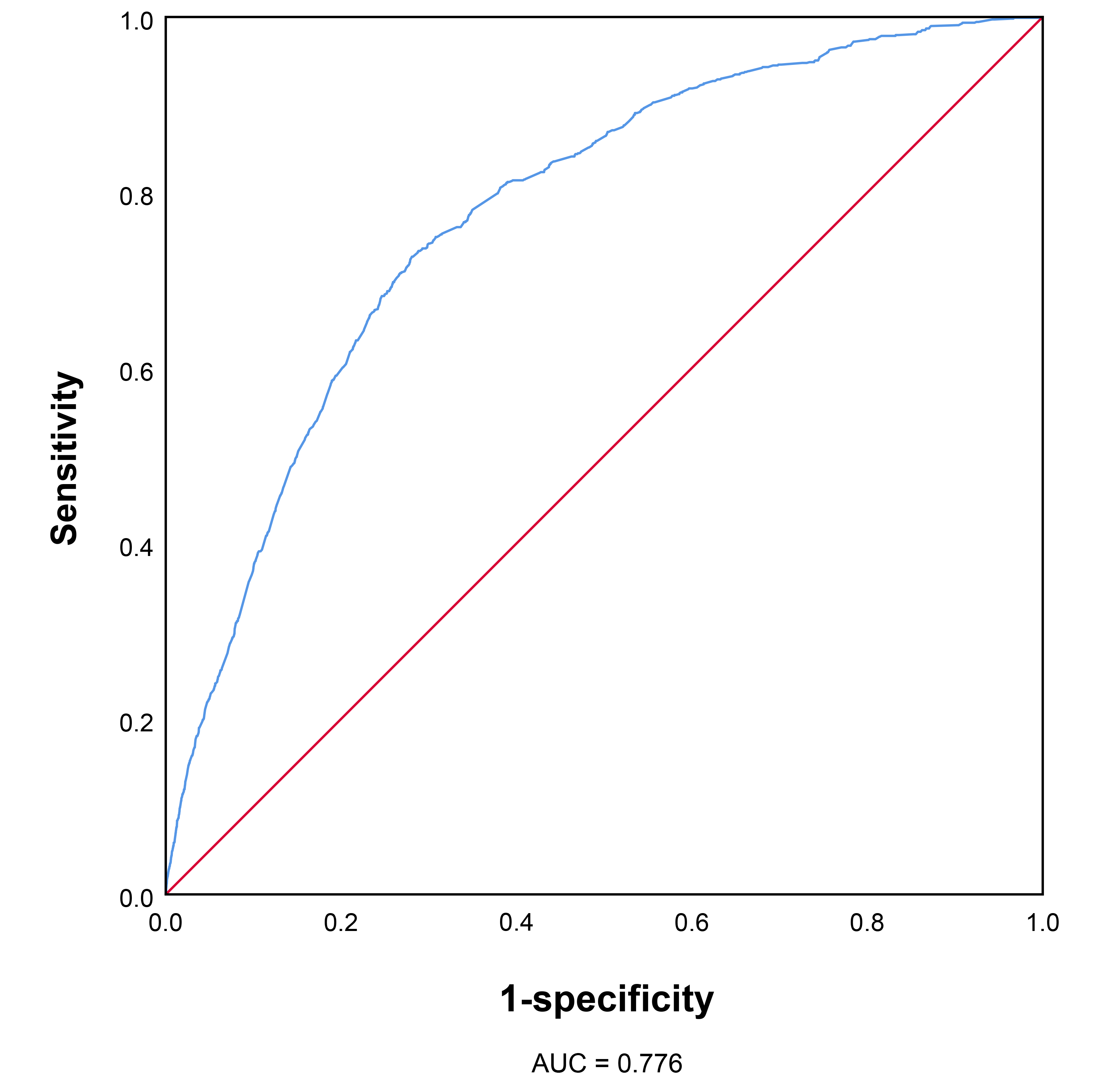

Supplement: Supplementary Figure 1 — The ROC curve and AUC of the risk factors in promoting distant metastasis in lymph-node-negative women. [file Image_1.tif]

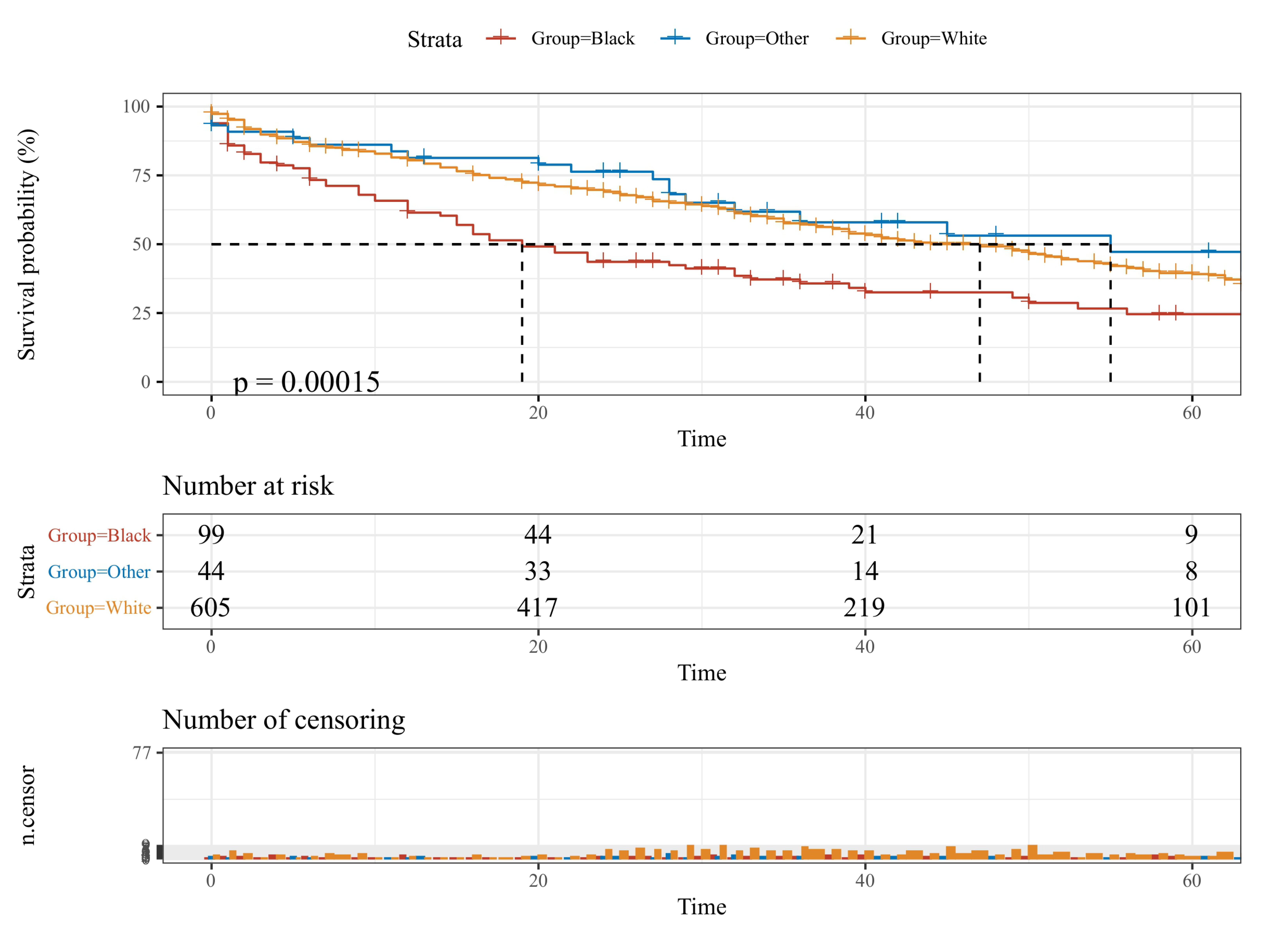

Supplement: Supplementary Figure 2 — The KM survival curves for predicting the CSS of lymph-node-negative women with DM according to race. [file Image_2.tif]

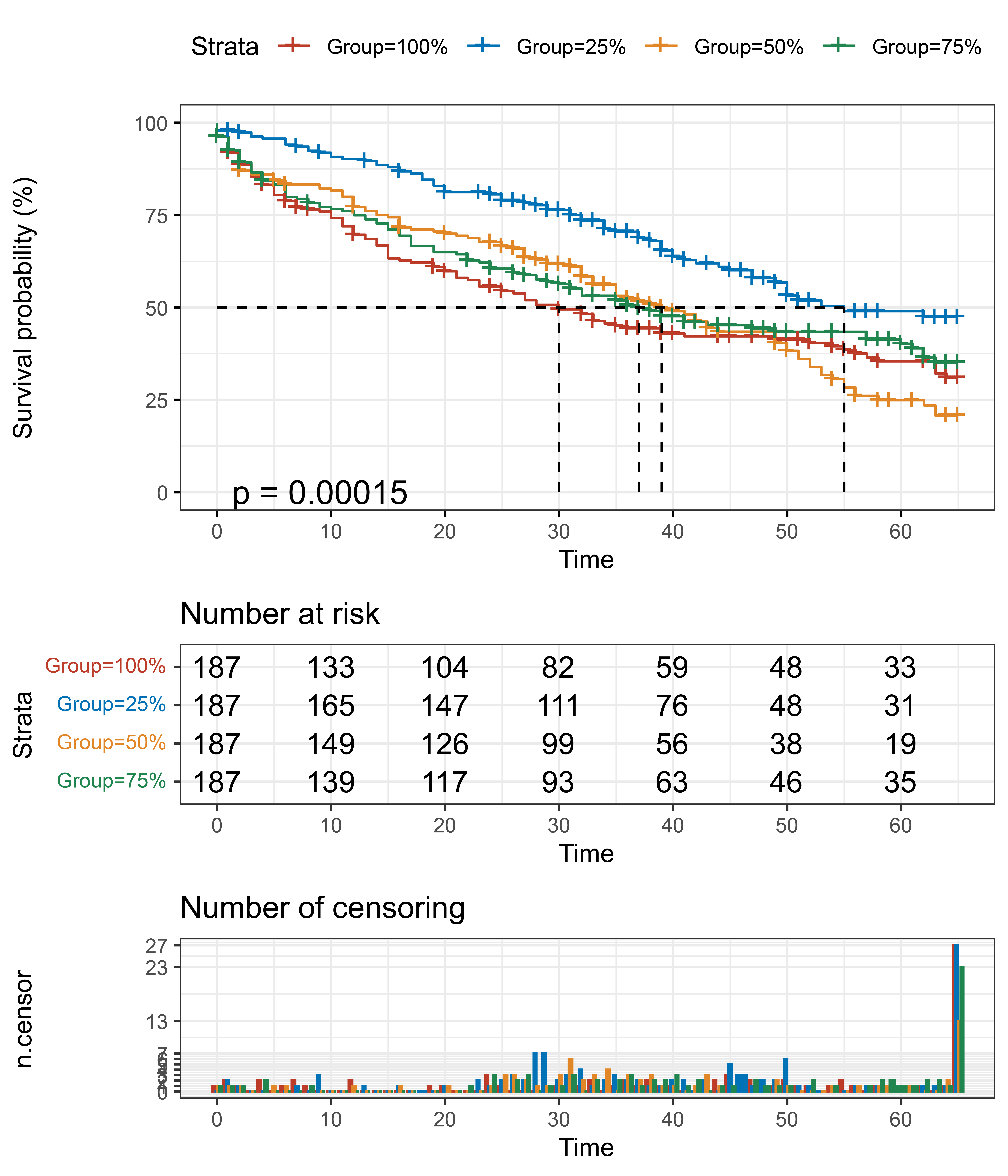

Supplement: Supplementary Figure 3 — The KM survival curves for predicting the CSS in the different risk population, based on the risk stratification of the nomogram. [file Image_3.tif]
